# Probabilistic risk assessment of dietary exposure to aflatoxin B_1_ in Guangzhou, China

**DOI:** 10.1038/s41598-020-64295-8

**Published:** 2020-05-14

**Authors:** Weiwei Zhang, Yufei Liu, Boheng Liang, Yuhua Zhang, Xianwu Zhong, Xiaoyan Luo, Jie Huang, Yanyan Wang, Weibin Cheng, Kuncai Chen

**Affiliations:** 1 Guangzhou Center for Disease Control and Prevention, Guangzhou, 510440 China; 2 Guangdong Second Provincial General Hospital, Guangzhou, 510000 China

**Keywords:** Environmental monitoring, Risk factors

## Abstract

Aflatoxin B_1_ (AFB_1_) contamination in foods is an important health challenge for low-and middle-income countries in subtropical regions. AFB_1_ has been detected in a variety of foodsin Guangzhou, while the risk of dietary exposure is unknown. This study aimed to assess the probabilistic risk of dietary exposure to AFB_1_ contamination in food stuffs in Guangzhou by using margin of exposure (MOE) and quantitative liver cancer risk approaches. A total of1854 AFB_1_-contaminated foodstuffs were sampled in supermarkets, agricultural markets, retail shops, and family workshops from 11 districts of Guangzhou, and AFB_1_ content was determined by HPLC-fluorescence detector. In total, 9.9% (184/1854) of the test samples had AFB_1_ concentrations above the limit of detection. Home-made peanut oil had the highest AFB_1_ concentration, with a mean value of 38.74 ± 47.45 μg kg^−1^. The average MOE levels of Guangzhou residents ranged from 100 to 1000. The risk of liver cancer was 0.0264 cancers (100,000 population year)^−1^. The health risks of suburban people were higher than those of urban people, and home-made peanut oil was the main contributorto dietary exposure to AFB_1_ among suburban residents in Guangzhou. The production of home-made peanut oil should be supervised to reduce the risk of AFB_1_ exposure.

## Introduction

Aflatoxins (AFs) are mycotoxins produced by the common fungi *Aspergillus flavus*and *Aspergillus parasiticus*^1^ and have been found in a wide range of crops such as maize, peanut, and walnut and their derived products^2^. There are four major aflatoxins (AFB_1_, AFB2, AFG1, and AFG2) produced by the two fungi that commonly found in contaminated crops^3–5^. AFB_1_ and AFB_2_ can be produced by *A. flavus* (both S and L strains) and *A. parasiticus*, whileAFG_1_ and AFG_2_ can be produced by *A. flavus* S strains and *A. parasiticus*^5,6^. AFB_1_ is considered the most toxic carcinogenwhich is classified as Group 1 human carcinogen by the International Agency for Research on Cancer (IARC)that induces mainly liver cancer^7–9^ and to a lesser degree rectal cancer^10^.

AFB_1_ is commonly found in cereals and nuts^11^, and it has attracted concern in lessdeveloped tropical regions^12–15^. Previous studies showed that AFs were found in 5%-30%of raw peanuts and peanut products in major peanut-producing regions in China^16^. Since some crops susceptible to AFs contamination, such as peanuts, are commonly consumed, it is hard to achieve zero exposure to AFs. Therefore, it is important to reduce the exposure to total AFsby establishing regulatory limits to AFs.The Codex Alimentarius Commission, the Joint Food and Agriculture Organization (FAO), and the World Health Organization (WHO) Food Standards Program jointly adopted a maximum level of 15 μg kg^−^1 for total AFsin unprocessed peanuts^17^. The European Commission regulation (EC) No. 1881/2006 set a maximum limit for AFB_1_ of 2 μg kg^−1^ for peanuts and cereals that are intended for direct consumption^18^. In China, the National Food Safety Standard set the limit of 20 µg kg^−1^ for AFB_1_ in peanut and its products and in maize and its products^19^. In addition to setting regulatory limits for AFB_1_,it is also necessary to conduct dietary exposure risk assessments in the population. A low-dose extrapolation approach introducedby the Joint FAO/WHO Expert Committee on Food Additives(JECFA)^20^ in 1997 and the margin of exposure (MOE) method proposed at the 64^th^ JECFAmeeting in 2005^21^ were both recommended and have been widely used worldwide^14,22,23^ to assess the risk of dietary exposure toAFB_1_.

JECFAperformed adietary exposure risk assessment for AFs as early as 1997. However, the data used were not considered representativebecause of the bias for the highest contamination level in food sampling^24^. In view of this scenario, national or regional AFhealth risk exposure assessments have beenundertakensince then,especially in tropical and subtropical regions^12–15^. In general, economically developed countries have a lower risk of health hazard assessment than developing or less developed countries. Dietary exposureto AFsestimated by the European Union ranged from 0.93 to 2.45 ng kg^−1^ bw day^−1^ for the lower bound to the upper bound^25^. In the United States, exposure was estimatedat 2.7 ngkg^−1^ bw day^−1^ ^26^ In Asia, the population of Japan^27^, with anintake ranging from 0.003 to 0.004 ngkg^−1^ bw day^−1^, hasa lower riskthan those of Indonesia^13^ (from 0.02 to 427.8 ngkg^−1^ bw day^−1^) and Vietnam^23^ (from 35.0 to 43.7 ngkg^−1^ bw day^−1^).

Guangzhou, one of the major metropolitan areas in southern China, is located in Guangdong Province and has more than 14 million people. Due to the subtropical monsoon climate (with a relatively humid environment), Guangzhou,with coexisting urban and rural areas, has been facing the challenge of AFB_1_contamination in foodstuffs^28–31^. Warm and humid conditions are favourable for *A.flavus* growth in some types of food, such as peanut and maize. A survey of foodstuffs (rice, wheat flour, peanut and peanut oil, corn flour and corn oil, and soybean) that areprone to contamination by AFs found that the overall detection rate of AFB_1_ was 31.7%, with the highest concentration of 39.3 µg kg^−^1 found in peanut oil^29^. However, the health risk of AFB_1_ dietary exposure to local residents was unknown.

In this context, Guangzhou Center for Disease Control and Prevention conducted a surveillance programme for three consecutive years to monitor contamination of foodstuffsby AFB_1_. We present the probabilistic risk of dietary exposure to AFB_1_ among Guangzhou residents by using MOE andquantitative liver cancer risk approaches.

## Materials and methods

### Sampling

From January 2015 to December 2017, typical AFB_1_-contaminated foodstuffs, includingrice and rice products, wheat and wheat products, maize and maize products, vegetable oil(including home-made peanut oil), nuts, and tea,were bought from household supply retail shops covered in all eleven districts of Guangzhou. These foods were considered theprobable sources of AFB_1_exposure in Guangzhou^29^.

Individual streetswere set as the sampling units. Street information was obtained from local governmental authorities. Three streets (two central streets and one remote street) were randomly selectedand stratified by district and type of streets (central or remote) using computer-generatedrandom digits. A total of 33 streets (22 central streets and 11 remote streets) were selected as food sampling sites. Trained investigators bought foodstuffs fromsupermarkets, agricultural markets, retail shops, and family workshops. Finally, a total of 1854 single-species food samples were included in this study, and alist of sampling sites isshown in Supplementary Table 1.

### Analytical procedure (high-performance liquid chromatography)

In accordance with a previouslyvalidated method, the procedure to determine AFB_1_in foods was applied with some slight modifications^32–34^. First,for solid samples, the sampling quantity should be more than 1 kg, and the sample should be crushed by a high-speed crusher and then sieved to make particles smaller than 2 mm. The test sieve should be mixed evenly, condensed to 100 g, and then stored in a sample bottle and sealed for storage until detection. The sampled amount of liquid samplesshould be greater than 1 L. For bagged, bottled and other packaged samples, at least 3 packages (the same batch or number) should be collected, all liquid samples should be mixed in a container with a homogenizer, and any 100 g (mL) of the liquid samples can be tested.The prepared samples were stored in a refrigerator at 0~4 °C for no more than 48 hours before analysis. Second,for solid samples, 5 g was weighed (accurate to 0.01 g) into a 50 mL centrifuge tube, 20.0 mL methanol-water solution (70 + 30) was added, and the sample was mixed by vortex, put into an ultrasonic oscillator for 20 min (or a homogenizer for 3 min), and centrifuged at 6000rmin^−1^ for 10 min (or applied to glass-fibre filter paper after homogenization).The supernatant was taken for later use.For vegetable oil, 5 g (accurate to 0.01 g) was placed into a 50 mL centrifuge tube, 20 mL methanol-water solution (70 + 30) was added, and the sample was mixed by vortex, put into ultrasonic oscillator for 20 min, and centrifuged at 6000rmin^−1^ for 10 min.The supernatant was taken for use.Whatman GF/A glass-fibre filter paper was used to filtrate 10 ml extract and to collect the filtrate in the clean container. In addition, 5 ml extract was diluted with 20 ml purified water and filtered before being tested. AFB_1_ content was determined by an HPLC-fluorescence detector (excitation, 360 nm; emission, 450 nm) (Waters Alliance e2695) by using post-column-photochemical reactor derivatization.

### Quality control

The limit of detection (LOD)in our study was0.1 μg kg^−1^ and determined from a signal-to-noise ratio equal to 3:1,and the limit of quantification(LOQ) was determined as the point at which this ratio was more than 10:1. Recovery rates in each foodwere ascertained by spiking with AFB_1_, and the rates rangedfrom 95% to 105%.

### Estimation of daily food consumption

Food consumption data were derived from a national food consumption survey of urban and rural residents in Guangzhou performed in 2011. Information on dietary intake was based on a three-day consecutive 24-h recall questionnaire in combination with the weighing method for edible cooking oil. Details of the methodology are available in our previously published manuscripts^35,36^. In sum, 2960 residents from 998 households were surveyed in the study. Among the subjects, 1416 were male and 1544 were female. Urban residents accounted for 63.8% (1888) of the total, and suburban residents accounted for 36.2% (1072). The age ranged from 3 to 86 years, and the mean age was 32 years. The age groupsof 3 to 6 years old, 7 to 17 years old, 18 to 59 years old, and 60 years old and above accounted for 6.7% (199), 21.5% (637), 58.6% (1734), and 13.2% (390) of the total people, respectively^16,37,38^.

In this study, vegetable oils collected in the survey included peanut oil, corn oil, tea seed oil, and soybean oil. According to the production conditions, vegetable oils were classified into commercial vegetable oil and bulk vegetable oil. Commercial vegetable oil was defined as the vegetable oil produced by licensed manufacturers and had underwent sampling inspection by the Chinese Food and Drug Administration. Bulk vegetable oil was produced by family workshops in the suburban areas, where this inspection usually did not in place. In this case, bulk vegetable oil referred to home-made peanut oil.

### Estimation of daily intake of AFB_1_

The total dietary intake of AFB_1_ was calculated as an estimateddaily intake (EDI) by using Eq. (1)^39^.

1 $$EDI=\mathop{\sum }\limits_{i=1}^{n}=\frac{Di\,\ast \,Mi}{W}$$

EDI was the estimation of daily dietary AFB_1_ intake (ng kg−1 body weight day−1). Di was the daily consumption of each food in each age group (gperson^−1^ day^−1^). Mi was the mean level of AFB_1_ in each food category (ngkg^−1^). When AFB_1_ was not detected in certain types of food, Mi was then assumed to be LOD/2^40^. The WHO recommended an alternative method to calculate the undetected value^41–43^. When the undetected samplevalueswere less than 60%, the non-detected value was replaced by the value of LOD / 2.When more than 80% of the sample values were not detected, the undetected valueswere replaced by 0 or LOD, respectively, as the lower bound and upper bound. However, in our study, the LODappears to be highcompared with those of other regions, such as Japan and Taiwan, China, where LODs ranged from0.001 to0.1 μg kg^−1^,but it was consistent withvalue in the Chinese National Food Contamination Monitoring Program.If the upper bound is used to estimate the mean value, the exposure risk might be overestimated. Our approach was to replace all the undetected values by LOD / 2, which ishighly conservative and doesn’t overestimatethe risk. W was the body weight of each respondent (kg). The average weight of respondents aged 3 to 6 years was determined to be 20 kg^44^, the average weight of respondents aged 7 to 17 years was determined to be 40 kg^45^, and members of the other two age groups were determined to average 60 kg^46^. Mean daily exposure to AFB_1_ was estimated by using the @RISK software.

### Risk characterization

#### Margin of exposure (MOE)

TheMOE method estimates the risk of genotoxic carcinogens^21^. It calculates the risk by the ratio of carcinogenic dose (or population carcinogenic dose) to population intake. The higher the MOE value is, the lower the risk of genetic carcinogen exposure.

MOE is calculated as the ratio between the points of departure (PODs) on the dose-response curve (animal or population carcinogenic dose) for a critical effect and the exposure level of the population. The formula is as follows: MOEs = PODs/dietary exposure (EDI). The European Food Safety Authority (EFSA) Scientific Panel on Contaminants in the Food Chain proposed the use of a benchmark dose lower confidence limit for 10% extra risk (BMDL10) and a benchmark dose lower confidence limit for 1% extra risk (BMDL1) for characterizing the MOE as PODs^25^. The value of BMDL10 (the lower limit for the 95% confidence interval of the 10% incidence of liver cancer in the control group) was 340 ng kg^−1^ bw day^−1^ for AFB_1_as referenced by the EFSA^25^. In reference to the dose-response relationship based on the data of Peers^47,48^ and Carlborg^49^, the value of BMDL1 (the lower limit for the 95% statistical confidence interval of the 1% incidence of liver cancer in the control group) was estimated as 78 ng kg^−1^ bw day^−1^. The reference value for a chronic dose that causes 25% of test animals to develop liver cancer (T25) during their standard lifespan was varied. The most widely used values were390 ng kg^−1^bw day^−1^(according to Benford^50^)and 500 ng kg^−1^ bw day^−1^(recommended by Wogen^51^). For safety considerations, we referred to the conservative T25 value of 390 ng kg^−1^bw day^−1^as the POD.

#### Quantitative risk assessment of liver cancer

Quantitative liver cancer riskassessment is one of the popular low-dose extrapolationapproaches used for AFB_1_ dietary exposure risk assessment. The low-dose extrapolation approachassumes that there is a linear dose-response relationship between the carcinogenic dose and the incidence of cancer in a population within a low-dose response range^15,52–54^. This methodtakes advantage of the exposure andpotency of carcinogens, providing quantitative data on human carcinogenic risk.This method was consistent with the formula proposed by the JECFA^20^. Because hepatitis B could synergistically increase the risk of AFB_1_-induced liver cancer, we separately estimated the carcinogenic potency in people who had hepatitis B and in peoplewho were hepatitis B negative. Studies have shown that the carcinogenic efficacy of AF in hepatitis B virus carriers is 30 times higher than that in nonviral carriers^27,53,55^. For hepatitis B surface antigen-positive individuals (PHBsAg + ), the potency was 0.3 cancers per year ng^−1^ AFB_1_ kg^−1^bw day^−1^per 100,000 population. For hepatitis B surface antigen-negative individuals (PHBsAg-), the potency was 0.01 cancers per year ng^−1^AFB_1_ kg^−1^bw day^−1^per 100,000 population^53,56,57^.

The cancer riskwas estimated by using Eq. (2).

2 $$\begin{array}{ccc}{P}_{{\rm{cancer}}} & = & ({\rm{PHBsAg}}\,+{\rm{xpop}}.{\rm{HBsAg}}\,+\,)+({\rm{PHBsAg}} \mbox{-} {\rm{xpop}}.{\rm{HBsAg}}-)\\ {\rm{Cancer}}\,{\rm{risk}} & = & {P}_{{\rm{cancer}}}\times {\rm{Estimated}}\,{\rm{Daily}}\,{\rm{In}}\,{\rm{take}}\,\end{array}$$

The prevalence of HBsAg+ was estimated to be 12.5% in Guangzhou, with 7.1% in urban areas and 16.1% in suburban areas^58^.

### Statistical analysis

Descriptive analysis was performed to describe the concentration of AFB_1_ in foodstuffs by using the mean ± standard deviation. Probabilistic risk assessment model calculations for AFB_1_ dietary exposure, MOE values, and cancer risk were performed by @RISK software (Palisade Corporation, 7.6. Industrial, 2018) based on a Monte Carlo simulation with 10000 iterations. The results are displayed as the mean values (range from the 5^th^ percentile to the 95^th^ percentile).

Due to the difference in vegetable oil consumption habits between urban and suburban residents, we conducted a sub-analysis by stratifying suburban residents from urban residents to see the difference in dietary exposure to AFB_1_. We found that the dietary intake of home-made peanut oil was reported among only suburban residents because such oil was predominantly sold in suburban areas.

## Results and discussion

### AFB_1_ levels in foods

**Figure 1:**
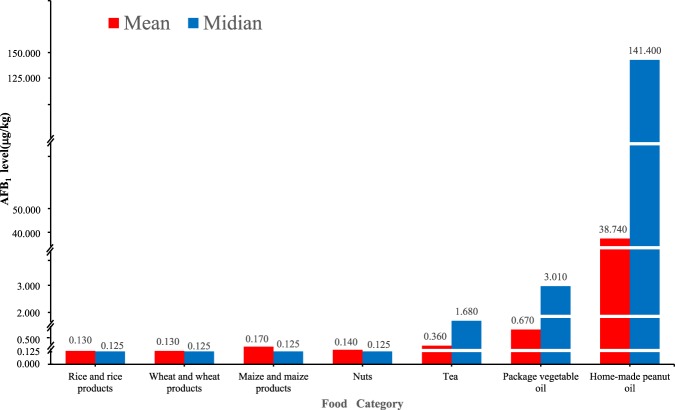
Aflatoxin B_1_ levels in seven kinds of foods in Guangzhou City.

The levels of AFB_1_ in 1854 food samples are summarized in Table 1. The levels of AFB_1_ levels in food samples between 2015, 2016, and 2017 were comparable (see Supplementary Table 2).The mean level of AFB_1_ in all samples was 1.4 μg kg^−1^, and the 50^th^ percentile (P50) and 95^th^ percentile (P95) values were not detected (ND) and 2.2 μg kg^−1^, respectively. In total, 9.9% (184/1854) of the test samples had AFB_1_ levels above the LOD. Home-made peanut oil had the highest concentration of AFB_1_, with detected values ranging from 0.26 to 283.0 μg kg^−1^, a median value of 3.21 μg kg^−1^ and a mean value of 38.74 ±47.45 μg kg^−1^. In rice and rice products, wheat and wheat products, maize and maize products, and nuts, AFB_1_ concentration levels were very low, and most results were under the detection limit (Fig. 1).

**Table 1:** AFB_1_ levels of foods in Guangzhou from2015 to 2017.

Food Category	Number of samples	<LOD	AFB_1_ level(μg kg^−1^)			
			Mean ±standard deviation	P50	P95	Range
Rice and rice Products	490	483	0.13 ± 0.001	ND	ND	0.28~1.00
Wheat and wheat products	436	430	0.13 ± 0.001	ND	ND	0.28~1.46
Maize and maize Products	339	336	0.17 ± 0.001	ND	ND	1.50~6.30
Nuts	96	93	0.14 ± 0.001	ND	ND	0.62~1.37
Tea	128	105	0.36 ± 0.62	ND	1.68	0.25~4.0
Vegetable oil ^a^	365^b^	223^e^	6.32±25.99	ND	30.45	0.26~283.0
1. Commercial vegetable oil	269^c^	201 ^f^	0.67 ±1.81	ND	3.01	0.35~7.30
2 Home-made peanut oil	**96**^**d**^	**22 **^**g**^	**38.74 ±47.45**	**3.21**	**141.40**	**0.26~283.0**
Total	1854	1670	1.40±11.94	ND	2.20	0.25~283.0

a = vegetable oil equalsthe sum of commercial vegetable oil and home-made peanut oil.

b = c+d.

e = f+g.

AFB_1_: Aflatoxin B_1_; LOD: Limit of detection; ND: Not detected.

Comparisonsof data from someSoutheast Asian countries show that the level of AFB_1_ contamination in some foods in Guangzhou, such as rice and maize, was relatively lower than that in some foods in Vietnam, where AFB_1_ contamination levels in maize, rice products and other cereals were 2.1~31.1 μg kg^−1^ ^59^, 2.7 μg kg^−1^ and 3.2 μg kg^−1^ ^23^, respectively. In addition, the contamination level of AFB_1_ in nuts (including peanut) was low in Guangzhou compared with other provinces of China^16^ and Malaysia (ranging from 0.40 μg kg^−1^ to 222 μg kg^−1^)^12^. However, the samples cited were raw peanut or maize samples, while in this study, all the samples were processed products. Generally, raw samples were relatively more contaminated with aflatoxins than were processed samples.For commercially processed samples, the levels of aflatoxin in peanut oil and maize were higher in Guangdong Province than in Fujian^60^ and Chongqing^61,62^. This situation promptedGuangzhouto pay attention to the contamination of AFB_1_ in peanut oil and find the source of the problem.

Our study is the first to include home-made peanut oil in the assessment of AFB_1_ in Guangzhou. The results showed that the alarmingly high AFB_1_ level in home-made peanut oil poses a potential public health threat among suburban residents in Guangzhou. Home-made peanut oil is widely consumed in many underdeveloped cities of China. People prefer home-made peanut oil because of traditional cooking styles and eating habits, particularly in rural areas^28^. Two factors might contribute to the contamination of home-made peanut oil by AFB_1_. One factor is that poor-qualityoil extraction machines and simple traditional procedures are unabletodegrade AFB_1_and that effective techniques to control AFB_1_ are difficult to apply in family workshops^63^. The other factor is that the peanuts used for oil extraction might becontaminated by AFB_1_ to different degrees.If the harvested peanuts that were not ready to be pressed soon for peanut oil were stored in a warm and humid environment,the AFB_1_ level could easily increase. If mouldy peanuts were not removed, the AFB_1_ level of peanut oil would hardly be reduced^28^.

Due to a lack of awareness of AFB_1_ contamination and the maximization of profits,oil mill ownerstend to use mouldy peanuts for oil extraction, which would not significantly affect the flavour of home-made peanut oil. This poor manufacturing practice is common because it is difficult for consumers to identify. Therefore, regulationand supervision of home-made peanut oil should be enhanced in Guangzhou.Findings from this study are also meaningful to regions where home-made peanut oilis widely available but whose production is unsupervised by food safety regulators.

### Dietary AFB_1_ exposure

The EDI of AFB_1_ in each AFB_1_-detected food in all age groups is presented in Table 2. The EDI in each AFB_1_-detected food for urban and suburban areas is presented in Table 3.In all age groups, the intake was the highest for rice and rice products among the contributed foods, and rice and rice product intake was the main contributor to AFB_1_ exposure in Guangzhou.Despite the low dietary consumption of vegetable oil, it was the second contributor due to its high AFB_1_ concentration. Wheat and wheat products were the third contributor to therelatively high consumption. However, theAFB_1_ concentration in wheat and wheat products was low. In addition, maize and maize products, tea, and nuts had little effect on the EDI due to their low consumption and AFB_1_ concentrations.

**Table 2:** Dietary consumption and AFB_1_ EDI for each AFB_1_-analysed food in different age groups in Guangzhou.

Food category	3~6years old		7~17years old		18~59years old		More than 60 years old		total	
	Dietary Consumption Reference Person± Standard Deviation (g day^−1^)	AFB_1_EDI (ngkg^−1^bwday^−1^)(90% confidence interval)	Dietary Consumption Reference Person± Standard Deviation (g day^−1^)	AFB_1_EDI (ng kg^−1^bwday^−1^)(90% confidence interval)	Dietary Consumption Reference Person±Standard Deviation (g day^−1^)	AFB_1_EDI (ng kg^−1^bwday^−1^)(90% confidence interval)	Dietary Consumption Reference Person± Standard Deviation (g day^−1^)	AFB_1_EDI (ng kg^−1^ bwday^−1^)(90% confidence interval)	Dietary Consumption Reference Person± Standard Deviation (g day^−1^)	AFB_1_ EDI (ng kg^−1^bwday^−1^)(90% confidence interval)
Rice and rice products	78.5 ± 45.5	0.50 (0.02~0.98)	121.3 ± 73.8	0.38 (0.01~0.77)	146.5 ± 90.5	0.31 (0.01~0.63)	126.5 ± 90.7	0.27 (0.05~0.58)	135.8 ± 86.9	0.29 (0.01~0.59)
Wheat and wheat products	32.6 ± 22.3	0.21 (0.03~0.44)	48.4 ± 38.2	0.16 (0.04~0.35)	52.3 ± 38.7	0.11 (0.02~0.25)	50.6 ± 33.1	0.12 (0.03~0.44)	49.3 ± 36.3	0.11 (0.03~0.27)
Maize and maize products	6.3 ± 4.8	0.04 (0.01~0.09)	7.0 ± 4.5	0.02 (0.01~0.04)	9.2 ± 8.7	0.02 (0.01~0.12)	14.7 ± 3.1	0.03 (0.01~0.03)	8.8 ± 5.3	0.02 (0.01~0.05)
Nuts	2.5 ± 1.9	0.02 (0.01~0.03)	2.2 ± 1.8	0.01 (0.01~0.02)	2.3 ± 2.2	0.01 (0.01~0.02)	1.14 ± 1.1	0.01 (0.01~0.02)	2.0 ± 1.9	0.01 (0.01~0.02)
Tea	0.1 ± 0.9	0.00 (0.00~0.02)	2.2 ± 1.5	0.01 (0.01~0.02)	4.5 ± 4.0	0.01 (0.01~0.05)	3.8 ± 3.1	0.01 (0.01~0.03)	3.6 ± 3.3	0.01 (0.01~0.03)
Vegetable oil	11.3 ± 9.6	0.17 (0.01~3.37)	22.3 ± 18.5	0.17 (0.01~3.31)	26.4 ± 17.3	0.13 (0.01~2.43)	9.6 ± 5.3	0.05 (0.01~0.88)	26.6 ± 16.8	0.13 (0.01~2.50)
Total		0.94 (0.29~4.24)		0.75 (0.22~3.64)		0.59 (0.20~3.11)		0.48 (0.16~1.41)		0.57 (0.21~3.16)

AFB_1_: Aflatoxin B_1_;EDI: Estimateddaily intake.

**Table 3:** AFB_1_ exposure in each AFB_1_-analysed food between urban and suburban areas in Guangzhou.

Food Category	Urban District		Suburban District	
	Dietary Consumption Reference Person (g day^−1^)	AFB_1_ EDI (ngkg^−1^bwday^−1^)	Dietary Consumption Reference Person (g day^−1^)	AFB_1_EDI (ngkg^−1^bwday^−1^)
Rice and rice products	118.5 ± 83.9	0.25 (0.04~0.54)	159.3 ± 90.1	0.33 (0.02~0.65)
Wheat and wheat products	51.6 ± 37.2	0.11 (0.02~0.24)	47.4 ± 35.3	0.10 (0.02~0.23)
Maize and maize Products	8.3 ± 5.8	0.02 (0.01~0.04)	9.0 ± 4.9	0.02 (0.01~0.04)
Nuts	2.5 ± 2.4	0.01 (0.01~0.03)	1.8 ± 1.7	0.01 (0.01~0.02)
Tea	3.5 ± 3.0	0.01 (0.01~0.03)	3.7 ± 3.2	0.01 (0.01~0.03)
Vegetable oil ^a^	25.9 ± 14.8	0.14 (0.01~0.37)	27.1 ± 18.1	1.78 (0.10~6.13)
1. Commercial vegetable oil	25.9 ± 14.8	0.14 (0.01~0.37)	25.0 ± 17.8	0.13 (0.00~0.38)
2. Home-made peanut oil	/		2.1 ± 1.95	1.65 (0.05~5.72)
Total		**0.29(0.08~0.56)**		**2.26(0.35~6.59)**

A: Total vegetable oil intake was equal to commercial vegetable oil plus home-made peanut oil (a = 1 + 2).

AFB_1_: Aflatoxin B_1_;EDI: Estimateddaily intake.

The EDI of AFB_1_ in each age group was estimated to range from 0.48 ng kg^−1^bw day^−1^ to 0.94 ng kg^−1^bw day^−1^, and the average EDI was estimated to be 0.57 ng kg^−1^bw day^−1^(the 90% confidence interval extended from 0.21 to 3.16). Among all age groups, the 3–6 years of age group had the highest EDI, with a value of 0.94 ng kg^−1^bw day^−1^. The difference in EDI between urban and suburban residents was large, with 0.29 ng kg^−1^bw day^−1^ and 2.26 ng kg^−1^bw day^−1^ for urban and suburban residents, respectively. The main source of suburban resident exposure to AFB_1_ was home-made peanut oil.

### Risk characterization using the MOE Approach

Table 4 presents the MOE values for AFB_1_ exposure. All MOE values were below the safe threshold of 10000. Probabilisticrisk analysis resultsshowed that most of the lower bound MOE values ranged from 10 to 100, indicating a concern for risk management.

**Table 4:** Risk characterization of AFB_1_ exposure in different age groups and different regions in Guangzhou based on the MOE approach.

Characteristic	POD(ng kg^−1^bwday^−1^)			Exposure (ng kg^−1^bwday^−1^)	MOE			
	T_25_	BMDL_10_	BMDL_1_		T_25_	BMDL_10_	BMDL_1_	
***Age group***	390	340	78					
3~6 years old				0.94 (0.29~4.24)	417(65~1086)	363 (62~931)		83 (14~215)
7~17years old				0.75 (0.22~3.64)	519 (65~1382	453 (51~1234)		104 (14~276)
18~59years old				0.59 (0.20~3.11)	654(96~1604)	570 (98~1478)		131 (20~347)
More than 60 years old				0.48 (0.16~1.41)	812(231~2199)	708 (204~1998)		162 (48~455)
***Region***								
Urban				0.29 (0.08~0.56)	1364 (657~4612)	1189 (573~4020)		273 (131~922)
Suburban				2.26(0.35~6.59)	172 (57~1055)	150 (50~920)		34 (11~211)
Total				0.57 (0.21~3.16)	681 (107~1719)	594 (88~1509)		136 (20~346)

AFB_1_: Aflatoxin B_1_; MOE: Margin of exposure; POD: Point of departure.

BMDL_10_: Benchmark dose lower confidence limit for 10%; BMDL_1_: Benchmark dose lower confidence limit for 1%; T_25_: The reference value of a chronic dose that causes 25% of test animals to develop liver cancer.

Age-group analysis suggestedthat we should pay close attention to the 3~6 years of age group, whose MOE value was the lowest. This result reflected that preschool children might have the highest risk of being exposed to AFB_1_.This agreed with the results from a studyfrom Taiwan in 2018 that found thatbabies and toddlers were at the highest risk of AFB_1_ exposure^64^.

Meanwhile, our results showed that the MOE value of suburban residents was lower than that of urban residents.AFB_1_ dietary exposure among urban residents in Guangzhou was similar to that of the urban residents in Shenzhen, an adjacent city to Guangzhou that is the most economically developed city in South China^38^. However, Guangzhou as a whole had a higher level of AFB_1_ risk than Shenzhen, probably because of the consumption of home-made peanut oil by suburban residents. In Guangxi Province, which neighbours Guangdong Province(where Guangzhouis located),grains and oil crops were also prone to mildew due to its subtropical climate with abundant year-round rainfall^63^. It should be noted that the residents of Guangxi Province have a similar habit of consuming home-made peanut oil. The mean AFB_1_ level of home-made peanut oil in the Guangxi study was 41.50 μg kg^−1^, slightly higher than the result in our study^35^. In a comparison of this study and studies from other low- and middle-income countries, dietary health risk exposure to AFB_1_ in Guangzhouappeared to be lower than that in other countries, and the risk of cancer was also lower than that in Indonesia^13^ and Vietnam^23^. The MOE values in our study were much greater than those in Japan^27^ and South Korea^22^,where socioeconomic statusisvery developed.

### Risk characterization using quantitative risk assessment of liver cancer

The potential cancer risk of AFB_1_ in Guangzhou residents was estimated by age group and by region (Table 5). In general, the risk of liver cancer in the entire population was estimated at 0.0264 cancers(year100 000 people)^−1^on average, which was far less than the incidence of liver cancer in China of 24.6 cancers(year 100 000 people)^−1^ ^65^. These results indicated that foods currently contaminated by AFB_1_ had low health risks for residents and that dietary exposure to AFB_1_may not account for the occurrence of liver cancer in Guangzhou.However, the EDI of suburban residents was nearly ten times higher than that of urban residents. The cancer risk among suburban residents was much higher than that among urban residents. These results were comparable to the results of a study conductedinGuangxi Province^66^, where dietary exposure to AFB_1_ was mainly caused by home-made peanut oil. Nonetheless, with the increasing vaccination rate for the hepatitis B vaccine in China, it is believed that the cancer risk will gradually decrease in the future.

**Table 5:** Estimated cancer risk in different age groupsand different regions in Guangzhou residents.

Characteristic	Exposure (ng kg^−1^bwday^−1^)	Fraction of population with hepatitis B	Annual hepatocellular carcinoma (HCC) incidence(cancers (year 100 000people)^−1^)
*Age group*			
3~6 years old	0.94(0.29~4.24)	12.45	0.0432(0.0001~1.4733)
7~17years old	0.75(0.22~3.64)		0.0346(0.0001~1.3403)
18~59years old	0.59(0.20~3.11)		0.0275(0.0001~0.8368)
More than 60 years old	0.48(0.16~1.41)		0.0221 (0.0001~0.4958)
*Region*			
Urban	0.29(0.08~0.56)	7.10	0.0088(0.0001~0.3507)
Suburban	2.26(0.35~6.59)	16.14	0.1284(0.0001~24.422)
**Total**	**0.57(0.21~3.16)**	**12.45**	**0.0264(0.0058~1.3802)**

### Uncertainty analysis and limitations

The entire process of food safety risk assessment has been accompanied by uncertainty. There are two main sources^67,68^. One source is extrapolation, wheredose levels inanimal studies exceed human exposure possibilities. Models used for extrapolation could cause results to differ by orders of magnitude, but uncertainty analysis can still improve transparency and assessment accuracy. The other source is data limitations, mainly including the inability to obtain theno observed adverse effect level(NOAEL), differences in exposure pathways, and differences in exposure time.Use of anuncertainty factor is a common method for dealing with these uncertainties^68^. Dividing the NOAEL obtained from animal experiments or other reference doses by theuncertainty factor can obtain a reference dose that is considered safe or without appreciable risk. The uncertaintyfactor is a coefficient that increases the level of protection of the health guidance value. The BMDL (with the uncertainty factor considered) used in the calculation of the exposure assessment in this study is a scientific method for dealing with data uncertainty.

Two factors need to be taken into consideration when these results are interpreted. First, AFs are jointly produced in nature, occurring as a mixture of AFB_1,_ AFB_2,_ AFG_1,_ AFG_2,_ etc.In our study, we assessed the risk of only AFB_1_, which wouldunderestimate the health risk of total AFs. However, AFB_1_ is the most toxic and frequent mycotoxinin AFs. Thus, the risk assessment forAFB_1_can reflect the overall risk of AFs. Second, although the consumption of home-made peanut oil among urban residents might be rare, the high concentration of AFB_1_ in home-made peanut oil requires the attention of the entire population. It would thus be necessary to expand the scale of home-made peanut oil consumption surveys to all residents instead of focusing on only suburban residents.

## Conclusions

This study is one of the few studies on probabilisticrisk assessment of dietary exposure to AFB_1_in China. Instead of studying the limited category of AFB_1_-contaminatedfood that is found in most studies, our study covered a wide variety of foods that mightcontribute to contamination byAFB_1_^16,64,69^. Thoughthe overall risk of dietary health risk exposure to AFB_1_ for liver cancer was low, there is a risk to health especially with continuous consumption. Furthermore, the health risk of suburban people was higher than that of urban people because of the common habit of consuming home-made peanut oil in the former group.In addition, 3~6-year-olds need special attention. Supervision of the production and sales of home-made peanut oil should be in place to reduce the risk of AFB_1_ exposure.

## Supplementary information

Supplementary information.
