# Supplementary material for: Probabilistic risk assessment of dietary exposure to aflatoxin B1 in Guangzhou, China
Source: Sci Rep. 2020 May 14;10:7973. doi: 10.1038/s41598-020-64295-8 (PMC7224072; doi:10.1038/s41598-020-64295-8)
Supplement: Supplementary file 1 — Supplementary information. [file 41598_2020_64295_MOESM1_ESM.docx]

**Probabilistic risk assessment of dietary exposure to aflatoxin B_1_ in Guangzhou, China**

Weiwei Zhang^1^, Yufei Liu^1^, Boheng Liang^1^, Yuhua Zhang^1^, Xianwu Zhong^1^, Xiaoyan Luo^1^, Jie Huang^1^, Yanyan Wang^1^, Weibin Cheng^2^ and Kuncai Chen^1^*

1. Guangzhou Center for Disease Control and Prevention, Guangzhou 510440, China;

2. Guangdong Second Provincial General Hospital, Guangzhou 510000, China;

gzcdczhangww@foxmail.com (W.Z.); gzliuyufei@hotmail.com (Y.L.); liangboheng-1999@163.com (B.L.); pisceszyh@126.com (Y.Z.); zhongxwgzcdc@foxmail.com (X.Z.); gzcdclxy@hotmail.com (X.L.); huangjie1026@126.com (J.H.), wangyy13213845@163.com (Y.W.); chwb817@gmail.com (W.C.)

***** Correspondence: ckc@gzcdc.org.cn; Tel.: +86-20-3605-5895

**Supplementary Table 1** Summary of food sampling sites

| **Food**  **category** | **Number of samples** | **Type of sampling site** | | | |
| --- | --- | --- | --- | --- | --- |
|  |  | supermarket | agricultural market | retail shop | family workshop |
| Rice and rice  products | 490 | 180 | 180 | 130 | 0 |
| Wheat and wheat products | 436 | 150 | 150 | 136 | 0 |
| Maize and maize  products | 339 | 120 | 113 | 106 | 0 |
| nuts | 96 | 30 | 30 | 36 | 0 |
| tea | 128 | 30 | 50 | 48 | 0 |
| package vegetable oil | 269 | 90 | 90 | 89 | 0 |
| home-made  peanut oil | **96** | **0** | **0** | **0** | **96** |
| Total | 1854 | 600 | 613 | 545 | 96 |

**Supplementary Table 2** Comparison of AFB_1_ levels in food samples collected in 2015, 2016, and 2017, in Guangzhou, China

| Food  Category | 2015 | | 2016 | | 2017 | | Kruskal-Wallis Test | |
| --- | --- | --- | --- | --- | --- | --- | --- | --- |
|  | Number of samples | AFB_1_ level | Number of samples | AFB_1_ level | Number of samples | AFB_1_ level | χ^2^ | *P* |
| Rice and rice  Products | 150 | 0.12 ± 0.001 | 150 | 0.13 ± 0.001 | 190 | 0.13 ± 0.001 | 2.37 | 0.305 |
| Wheat and wheat products | 145 | 0.13 ± 0.001 | 150 | 0.12 ± 0.001 | 141 | 0.13 ± 0.001 | 4.21 | 0.122 |
| Maize and maize  Products | 99 | 0.15 ± 0.001 | 100 | 0.18 ± 0.001 | 140 | 0.17 ± 0.001 | 5.38 | 0.107 |
| Nuts | 30 | 0.14 ± 0.001 | 30 | 0.14 ± 0.001 | 36 | 0.14 ± 0.001 | 1.89 | 0.504 |
| Tea | 60 | 0.40 ± 0.59 | / |  | 68 | 0.32 ± 0.67 | 3.96 | 0.076 |
| Vegetable oil^a^ |  |  |  |  |  |  |  |  |
| 1.Commercial vegetable oil | 70 | 0.77±1.23 | 99 | 0.69 ±1.93 | 100 | 0.63 ±1.88 | 5.73 | 0.085 |
| 2.Home-made  peanut oil | 30 | 42.56 ±48.02 | 35 | 39.71 ±45.34 | 31 | 34.88 ±51.73 | 6.02 | 0.061 |
| Total | 584 |  | 564 |  | 706 |  |  |  |

a=vegetable oil equals the sum of commercial vegetable oil and home-made peanut oil
